# Scope, Findability, and Quality of Information About Music-Based Interventions in Oncology: Quantitative Content Analysis of Public-Facing Websites at National Cancer Institute–Designated Cancer Centers

**DOI:** 10.2196/53440

**Published:** 2024-11-22

**Authors:** Carol Ann Blank, Sarah Biedka, Abigail Montalmant, Katelyn Saft, Miranda Lape, Kate Mao, Joke Bradt, Kevin T Liou

**Affiliations:** 1 Music, Creativity and Wellness Lab, College of Nursing and Health Professions, Drexel University, Philadelphia, PA, United States; 2 The City University of New York School of Medicine, New York, NY, United States; 3 Hunter College High School, New York, NY, United States; 4 Integrative Medicine Service, Bendheim Integrative Medicine Center, Memorial Sloan Kettering Cancer Center, 321 East 61st Street, 4th Floor, New York, NY, 10065, United States, 1 646-608-8558, 1 212-717-3185

**Keywords:** music-based interventions, cancer, oncology, symptom management, music therapy, music services, National Cancer Institute

## Abstract

**Background:**

Music-based interventions (MBIs) are evidence-based, nonpharmacological treatments that include music therapy (MT) delivered by board-certified music therapists, as well as music services (MS) delivered by other health professionals and volunteers. Despite MBI’s growing evidence base in cancer symptom management, it remains unclear how MBI-related information is presented to the public. Over 80% of people with cancer use the internet to find health-related information. In the United States, the National Cancer Institute (NCI) identifies certain Cancer Centers (CCs) as NCI-designated CCs or Comprehensive Cancer Centers (CCCs) based on their excellence in research. As NCI-designated CCs and CCCs are considered the gold standard in cancer care, their websites are viewed by the public as important sources of information.

**Objective:**

We aimed to determine scope, findability, and quality of MBI-related information on public-facing websites of NCI-designated CCs/CCCs.

**Methods:**

We reviewed 64 NCI-designated CC/CCC websites (excluding basic laboratories) between November 2022 and January 2023. We extracted data on the scope of information: (1) type of MBI offered (MT or MS), (2) format (individual, group), (3) method of delivery (in person or remotely delivered), (4) setting (inpatient or outpatient), (5) target population (pediatric or adult), (6) MBI practitioner qualifications, (7) clinical indications or benefits, (8) presence of testimonials, (9) cost, and (10) scheduling or referral information. We also extracted data on findability (ie, presence of direct link or drop-down menu and the number of clicks to locate MBI-related information). Based on the scope and findability data, we rated the information quality as high, moderate, or low using an adapted scale informed by prior research.

**Results:**

Thirty-one (48%) of the 64 CC/CCCs described MBIs on their websites. Of these, 6 (19%) mentioned both MT and MS, 16 (52%) mentioned MT only, and 9 (29%) mentioned MS only. The most common format was hybrid, involving individuals and groups (n=20, 65%). The most common delivery method was in person (n=16, 52%). The most common target population was adults (n=12, 39%). The most common MBI practitioners were board-certified music therapists (n=21, 68%). The most described indications or benefits were psychological. Twenty-eight (90%) websites lacked testimonials, and 26 (84%) lacked cost information. Twenty-six (84%) websites provided scheduling or referral information. MBI-related information was found with an average of 4 (SD 1) clicks. Nine (29%) websites were of high quality, 18 (58%) were moderate, and 4 (13%) were low.

**Conclusions:**

Based on public websites, MBIs were most commonly delivered in person by board-certified music therapists to outpatient and inpatient adults, using individual and group formats to provide psychological benefits. The findability and quality of this information should be improved to promote the dissemination of MBIs for cancer symptom management.

## Introduction

Management of symptoms and treatment-related side effects are a key priority in oncology [1]. Undertreatment of symptoms may contribute to worse cancer-related outcomes, including treatment nonadherence, higher health care use, and increased mortality [2-9]. Medications are commonly used for managing symptoms. However, polypharmacy represents a major concern in the cancer population, with approximately 64% already taking five or more medications [10]. Polypharmacy is associated with financial toxicity, higher risk of side effects and adverse medication interactions, and poor quality of life [10,11]. These risks of polypharmacy underscore the need for effective nonpharmacological approaches for cancer symptom management [11-13]. Various nonpharmacological interventions (eg, exercise and cognitive-behavioral therapy) have demonstrated effectiveness for cancer-related symptoms, such as fatigue or psychological distress [14,15], but these options may not be optimal for all individuals. For example, barriers to physical activity are well documented in cancer populations [16]. Furthermore, some cultures view conventional psychotherapy, such as cognitive behavioral therapy, as stigmatizing [17,18]. Due to these limitations, there is a critical need for more nonpharmacological options to address the diverse needs of patients with cancer.

Music-based interventions (MBIs) are evidence-based, nonpharmacological treatment options that include music therapy (MT) and music services (MS) [19,20]. MT is delivered in individual or group-based formats by board-certified music therapists who guide patients through music experiences to achieve therapeutic goals. These music experiences may include listening to live, improvised, or prerecorded music; playing instruments; improvising music using voice or instruments; and songwriting [20]. Board-certified music therapists are trained to design and facilitate personalized therapeutic processes to address individual needs [21]. In contrast to MT, MS are not delivered by board-certified music therapists; MS is a broad category that includes music performances by volunteer musicians in medical settings and listening to prerecorded music offered by medical personnel [22].

MBIs have a robust evidence base for cancer symptom management [20], particularly for symptoms that have been identified by the National Cancer Institute (NCI) as high priority [1]. A recent Cochrane review found that MBIs demonstrated effectiveness for anxiety, depression, pain, fatigue, and quality of life [20] As a result, MBIs are recommended in several clinical guidelines for cancer symptom management [23-26]. In a recently published joint guideline from the Society for Integrative Oncology and the American Society of Clinical Oncology, MT was recommended for anxiety and depression during active cancer treatments [25]. MBIs are thought to improve cancer-related symptoms through music’s effects on brain regions (eg, amygdala), psychosocial processes, as well as biological systems (eg, hypothalamic-pituitary-adrenal axis, autonomic nervous system) [27-31]. Given that music is a potent inducer of reward responses [32-34], MBIs could also potentially be more engaging for patients with cancer who find it difficult to adhere to other nonpharmacological interventions. Finally, music is found in nearly all cultures around the world [35,36]. This multicultural presence supports the unique potential of MBIs to appeal to diverse cancer populations as an option for symptom management.

Despite the growing evidence of MBIs, the availability of information about MBIs in the public sphere remains unclear. Gaps in knowledge about MBIs could limit the adoption and uptake of this evidence-based modality by patients, families, and their health care providers. One study found that most people search for health-related information online, and nearly 60% experience frustration during the online search process [37]. Another study found that over 80% of people with cancer use the internet to find health-related information [38], and they most commonly search for information related to treatment options and complementary therapies [39].

In the United States, NCI identifies certain Cancer Centers (CCs) around the country as NCI-designated CCs or NCI-designated Comprehensive Cancer Centers (CCCs) based on their scientific excellence and research capacities. NCI-designated CCs demonstrate excellence in laboratory, clinical, or population science research, whereas NCI-designated CCCs meet additional rigorous standards, including greater depth and breadth of research, access to more extensive resources, and greater transdisciplinary collaboration across basic, clinical, and population science research. Since NCI-designated CCs and CCCs are considered the gold standard in cancer care, their websites are often viewed as an important resource for health-related information. However, prior research has demonstrated key information gaps on these websites for topics relevant to patients with cancer [40,41]. Therefore, we investigated the scope, findability, and quality of MBI-related information on the public-facing websites of NCI-designated CCs and CCCs.

## Methods

### Study Design

This study is a quantitative content analysis of information about MBIs found on public-facing web pages of NCI-designated CCs [42,43]. Content analysis is a systematic method to code and quantify written, visual, or oral content [44]. For ease of reading, we will use the term “CC” to refer to both CCs and CCCs, except in instances where the distinction between CCs and CCCs is important to highlight.

### Inclusion and Exclusion Criteria for Websites

Websites were included in this study based on these criteria: (1) they belonged to an NCI-designated CCC or an NCI-designated CC. and (2) they contained MBI-related information. Websites were excluded from this study based on this criterion: (1) they were from an NCI-designated CC that was categorized as a basic laboratory.

### Search Strategy

**Figure 1.**
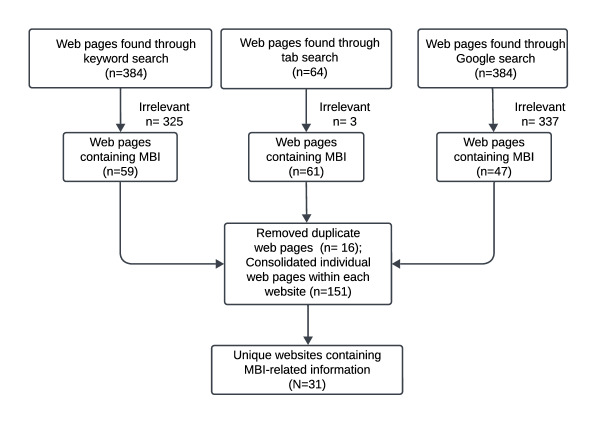
Search strategy for identifying National Cancer Institute–designated Cancer Center websites containing information related to music-based interventions.

Six members of our research team executed the search strategy and data extraction (CAB, SB, AM, KS, ML, and KM). These team members included a postdoctoral researcher, as well as high school, undergraduate, and graduate students. Data extraction was completed in pairs with one person handling the initial data extraction and the second person checking the data extraction for accuracy. We reviewed 64 NCI-Designated CCs listed on the NCI website [45] between November 2022 and January 2023, excluding the seven CCs identified as basic laboratories. Following the methods from similar studies, [46,47] we used three different search strategies to identify CCs that offer MBIs: (1) keyword searching with search terms “music,” “music therapy,” “musician,” “therapeutic music,” “harp,” and “sing” using the CC website’s search function; (2) tab searching, which entailed systematically reviewing each menu tab or link on the website’s home page (eg, “patient information” tab or “services” tab) for MBI-related information; and (3) the first page of results from Google searching using the aforementioned search terms combined with the name of the CC (Figure 1). Mentions of music that were irrelevant to MBI (eg, music fundraisers) were removed. Duplicate web pages were also removed. Individual web pages within each specific website were consolidated. Two coders used each search strategy. An Excel (Microsoft Corp) spreadsheet was used for data abstraction, with a priori determined categorical data entries as well as open text fields to allow for entry of more detailed descriptions. The team met biweekly to peer-check the coding and resolve search discrepancies through discussion.

### Evaluating the Scope of MBI-Related Information

We extracted the following MBI-related information from the public-facing websites of NCI-designated CCs: (1) type of MBI offered (eg, MT or MS) and the specific music activities involved (eg, music listening or group drumming), (2) format (eg, individual or group), (3) method of delivery (eg, in person or remotely delivered), (4) setting (eg, inpatient or outpatient), (5) target population (eg, pediatric or adult), (6) MBI practitioner qualifications (eg, board-certified music therapist, volunteer musician, etc), (7) clinical indications or treatment benefits (eg, reducing anxiety), (8) presence of testimonials about MBIs, (9) cost or fees, and (10) information about scheduling MBIs or referring patients for MBI.

We used the Theory of Planned Behavior (TPB) to inform our data extraction [48]. The TPB posits that an individual engages in a specific health behavior (eg, use of MBI) as a result of three key factors: (1) expected benefits or outcomes of the behavior (eg, symptom burden reduction), (2) perceived barriers to engaging in the behavior (eg, unfamiliarity or lack of knowledge about what MBIs entail, high costs associated with MBI use, limited availability of qualified MBI providers), and (3) social norms regarding the behavior (eg, information regarding for which patient populations MBIs are intended, testimonials from other patients with cancer, and recommendations from oncologists). Prior research demonstrated that these TPB factors predict the use of complementary alternative medicine by patients with cancer [49,50]. Thus, by extracting information across the three TPB domains, we were able to not only capture the scope of MBIs offered at CCs but also determine whether websites contain the critical information that influences patients’ decision to seek MBIs.

### Evaluating Findability of MBI-Related Information

Given that over 60% of patients experience frustration while searching for health-related information online [38,39], we evaluated findability of MBI-related information by first identifying CCs with a website home page that contained an easily identifiable link or drop-down menu to direct patients to MBI-related information. For CCs that did not contain an easily identifiable direct link or drop-down menu, we quantified the success path, a common website navigation metric, which we define here as the number of clicks needed to reach MBI-related information from a CC’s home page [51,52]. Similar approaches have been used in other research to evaluate the findability of information on CC websites [41].

### Evaluating Quality of MBI-Related Information

We used the scope and findability of MBI-related information to assign an overall information quality rating for each CC website (Table 1). Our scale for rating the quality of MBI-related information was adapted from the approach used by Silver et al [41] who similarly used findability and scope of information to assign a quality rating.

**Table 1.** Quality rating scale for information about music-based interventions found on National Cancer Institute–designated Cancer Center websites.

Rating	Findability	Scope of information provided
High	Success path ≤3 clicks	Web pages provide the following information: (1) type of music-based interventions offered; (2) clinical indications or treatment benefits; and (3) at least two of the following: (a) practitioner qualification, (b) referral information, (c) cost of service, (d) testimonials, (e) video of music-based intervention patient encounter, and (f) research evidence.
Moderate	Success path >3 clicks	Web pages provide the following information: (1) types of music-based interventions offered; (2) clinical indications or treatment benefits; and (3) only one of the following: (a) practitioner qualification, (b) referral information, (c) cost of service, (d) testimonials, (e) video of music-based intervention patient encounter, and (f) research evidence.
Low	Success path >5 clicks or no success locating the information with a keyword search	Web pages state that music-based interventions are offered but contain no details regarding type of music-based interventions offered; clinical indications or treatment benefits; practitioner qualifications; referral information; cost of service; testimonials; video of music-based intervention patient encounter; or research evidence.

### Ethical Considerations

This study analyzed publicly available information from websites in the public domain. No human subjects were involved in this study, and no personal identifiers or confidential data were collected. Therefore, approval from an institutional review board was not required for this type of research.

## Results

### Characteristics of CCs With MBIs

Of the 64 CCs, we identified 31 (48%) CCs that had information about MBIs on their public-facing website. Table 2 summarizes the NCI designation and regions for the 31 CCs that offered MBIs at the time of data collection. Links to the websites of these CCs can be found at this NCI directory [45].

**Table 2.** Characteristics of 31 Cancer Centers mentioning music-based interventions on their websites.

Characteristics	Cancer Centers, n (%)
National Cancer Institute–designation	
Cancer Center	8 (26)
Comprehensive Cancer Center	23 (74)
Region	
Midwest	5 (16)
Northeast	7 (23)
Southeast	9 (29)
Southwest	3 (10)
West	7 (23)

### Scope of Information About MBIs

#### Overview

The scope of information about MBIs found on the NCI-designated CC websites is summarized in Table 3.

**Table 3.** Scope of information about music-based interventions presented on the 31 National Cancer Institute–designated Cancer Center websites.

Characteristics	Cancer Center websites, n (%)
Type of music-based intervention	
Music therapy only	16 (52)
Music services only	9 (29)
Music therapy and music services	6 (19)
Format of music-based intervention	
Individual	2 (6)
Group	4 (13)
Both	20 (65)
Not reported	5 (16)
Method of delivery	
In person	16 (52)
Telehealth	2 (6)
Both	6 (19)
Not reported	7 (23)
Setting	
Inpatient	5 (16)
Outpatient	1 (3)
Both	19 (61)
Not reported	6 (19)
Target population	
Adult	12 (39)
Pediatric	5 (16)
Both	10 (32)
Not reported	4 (13)
Music-based intervention practitioners	
Board-certified music therapists	21 (68)
Other health professional	1 (3)
Musician	4 (13)
Other volunteers	5 (16)
Patient testimonials	
Yes	3 (10)
No	28 (90)
Cost or fee	
Free	2 (6)
Partially funded	3 (10)
No information provided	26 (84)
Scheduling or referral information	
Yes	26 (84)
No	5 (16)

#### Types of MBIs

Sixteen (52%) CCs listed MT services on their websites and did not appear to offer MS by nonmusic therapists. Nine mentioned only MS. Six CCs offered both MT and MS. Twenty-eight (90%) CCs provided information about the specific activities involved with MBIs. We grouped these activities into broad categories, such as listening to prerecorded music, songwriting, and lyric discussion (Table 4). The most frequently offered activities were music improvisation, playing instruments, music-guided relaxation, and songwriting.

**Table 4.** Specific music-based intervention activities described on National Cancer Institute–designated Cancer Center websites.

Interventions	Music therapy programs (n=23), n (%)	Music services programs (n=14), n (%)
Listening to prerecorded music	11 (48)	0 (0)
Music-guided relaxation	18 (78)	5 (36)
Singing	14 (61)	1 (7)
Lyric discussion	12 (52)	0 (0)
Music-guided movement	5 (22)	0 (0)
Learning or performing music	6 (26)	0 (0)
Music improvisation or playing instruments	21 (91)	2 (14)
Music making with family	5 (22)	0 (0)
Recording legacy music	6 (26)	0 (0)
Songwriting	18 (78)	0 (0)
None listed	2 (9)	1 (7)
Other^a^	7 (30)	9 (64)

a Includes listening to music in public spaces, neurologic music therapy, nonspecified music-based practices, and watching music videos.

#### Format

Twenty (65%) CCs offered MBIs in both individual and group formats. Two (6%) CCs only offered MBIs in an individual format; four (13%) CCs offered only group sessions. Five (16%) CCs did not report format information.

#### Method of Delivery

Sixteen (52%) CCs offered MBIs only in person. Six (19%) CCs offered both in-person and remotely offered MBIs. Two (6%) CCs offered MBIs exclusively through remote means. Seven (23%) CCs did not report on the method of delivery.

#### Setting

Nineteen (61%) CCs offered MBIs to inpatients and outpatients. Five (16%) CCs reported offering MBIs only in the inpatient setting; one (3%) CC reported offering MBIs only in the outpatient setting. Six (19%) CCs did not report this information.

#### Population

Five (16%) CCs offered MBIs to pediatric patients, 12 (39%) offered MBIs to adult patients, and 10 (32%) offered MBIs to both pediatric and adult patients. Four (13%) CCs did not specify the population.

#### MBI Practitioner Qualifications or Backgrounds

Twenty-one (68%) CCs had board-certified music therapists on staff. Four (13%) CCs had MBI programs that were staffed with a musician. One (3%) CC reported MBIs were provided by other health professionals. Five (13%) CCs were staffed with volunteers (including medical students). Only one (3%) CC described a relationship with a national organization that trained volunteers to provide MBIs to hospitalized patients [53]. All other centers lacked information about the type of training provided to volunteers delivering MBIs.

#### Clinical Indications and Treatment Benefits

Table 5 summarizes MBI treatment benefits or clinical indications described on CC websites. Due to the wide range of described benefits and indications, we grouped them into broad categories: physical (eg, symptom management, physical rehabilitation, or procedural support), psychological or emotional (eg, mood or coping, relaxation, quality of life, grief, or medical trauma), spiritual (eg, spirituality), and social (eg, communication, familial or care bond, or sense of community). Only two (6%) CCs offered a summary of research evidence for MBIs.

**Table 5.** Treatment benefits or clinical indications of music-based interventions described on National Cancer Institute–designated Cancer Center websites.

Treatment benefit or clinical indication	Music therapy programs (n=23), n (%)	Music services programs (n=14), n (%)
Physical	18 (78)	4 (29)
Psychological	21 (91)	11 (79)
Spiritual	6 (26)	2 (14)
Social	18 (78)	5 (36)

#### Patient Testimonials

Patient testimonials about treatment benefits were included on three (10%) web pages. We found a single testimonial by a health care provider on one (3%) website and one (3%) by a caregiver on another website. Twelve (39%) CCs provided video examples of patient encounters with MBIs.

#### Costs

Most of the websites did not include information about costs or fees associated with MBI services. Three (10%) websites stated MBIs were partially funded. Only two (6%) websites specified that MBIs were offered free of cost.

#### Scheduling and Referrals

Of the 31 CCs that offered MBIs, 26 (84%) provided referral information. MT was available via clinician referral or patient self-referral at 19 (61%) CCs. Eight (26%) websites instructed patients to contact the department responsible for offering MS.

### Findability of MBI-Related Information

Of the 31 CCs that offered MBIs, none (0%) had a tab or link on their home page that led directly to information about MBI. Relevant information about MBIs was found with an average of 4 (SD 1) clicks for 29 (94%) websites. Tab searching for MBIs was unsuccessful for two (6%) CCs. The success path for finding information about MBIs through tab searching took many forms but most often started in the “for patients” tab on the home page, then to “support services,” “integrative services,” or “rehabilitative services” tabs where MBI-related links were often present. Information regarding MBIs was sometimes found in additional tabs such as “child life,” “creative arts,” “palliative care,” or “wellness.” Keyword searching using the home page search function led to successful identification of information about MBIs for 28 (90%) CCs. For three (10%) CCs, keyword searching yielded marketing or media stories and event calendars but no further information about the types of MBIs provided or how to access them. Finally, Google searching (entering the name of the CC and the aforementioned keywords) produced MBI-related search results for 29 (94%) CCs. However, Google searching often produced results irrelevant to finding center-specific MBI-related information, such as benefit concerts, news reports, fundraising events for the hospital, or outdated promotional materials.

### Quality of MBI-Related Information

Using our quality rating scale (Table 1), we found that 9 (29%) CCs qualified for a high rating, 18 (58%) for a moderate rating, and 4 (13%) for a low rating.

## Discussion

### Principal Results

People with cancer often experience high symptom burden and are increasingly turning to the internet to find treatment options [37,54]. MBIs have a growing evidence base for cancer symptom management and are currently offered in various oncology settings [19,20,25,26,46,47,55], but there is a paucity of research on what types of MBI-related information are available on the internet. This is the first comprehensive study to examine the scope, findability, and quality of MBI-related information on the public-facing websites of NCI-designated CCs.

At the time of this study, the public-facing websites of 31 of 64 NCI-designated CCs offered information about MBIs at their respective CC. MBIs described on the CC websites varied widely by activities offered, practitioner, format, target population, and settings. These findings highlight the clinical versatility and adaptability of MBIs but also underscore the need for developing strategies and resources to help patients and families navigate the wide range of MBI-related options.

While the evidence base for MBIs continues to grow, additional research is still necessary to better understand the role of MBIs in cancer symptom management. Most websites indicated that MBIs improve outcomes in physical, psychological, spiritual, and social domains. Evidence from recent systematic reviews demonstrate that MBIs improve some physical (eg, pain or fatigue) and psychological (eg, anxiety or quality of life) outcomes listed on the websites [20,55]. However, several listed outcomes related to physical (eg, respiratory outcomes) and psychological (eg, sense of self, bereavement, self-expression, or executive function skills) domains are not yet supported by randomized controlled trials in oncology. Some CC websites also claimed that MBIs can improve outcomes in social (eg, familial bonds) and spiritual domains. These claims, however, are not yet supported by research evidence. Further, only two websites supported their stated treatment benefits with research evidence. As patients attempt to make informed treatment decisions for cancer symptom management, it is important for CCs to present accurate, evidence-informed information about the treatment benefits and clinical indications of MBIs in oncology.

At most centers, MBIs were provided by board-certified music therapists. These centers may additionally have volunteers, musicians, or other nonmusic therapist health care professionals who offered MS to patients such as listening to prerecorded or live music. Offering MS can increase access to MBIs, particularly in settings with limited availability of board-certified music therapists. However, prior research has shown that MS may produce inconsistent benefits relative to MT [19,20]. Furthermore, without proper guidance from a trained therapist, musical engagement can evoke strong emotions and memories that are undesirable or even harmful to people with cancer [19,56]. Public-facing websites often neglected to mention these risks when offering services by nonmusic therapists. Greater emphasis should be placed on educating patients about the distinction between MT and MS, as well as their relative benefits and risks.

The COVID-19 pandemic has accelerated the adoption of telehealth. However, only eight centers mentioned remotely delivered MBIs as an option on their websites. A growing body of research has documented that people with cancer experience time toxicity, which is conceptualized as the substantial amount of time spent in coordinating medical care, undergoing tests and treatments, and traveling to and from in-person appointments [57-63]. Offering more remotely delivered MBI options and emphasizing online services on CC websites may reduce time toxicity barriers and encourage patients with cancer to seek supportive care in the form of MBIs.

In parallel to increasing adoption of telehealth services, patients and families are more frequently turning to online sources for health-related information. Despite these trends in digitalization, the paucity of direct links to MBI-related information may result in fragmentation of information across multiple pages, increasing frustration for people who search online for MBI information. Furthermore, 22 CCs received a moderate or low rating for quality of MBI-related information, indicating that key pieces of information were lacking on their public-facing websites. For example, four or more websites lacked information on MBI format, delivery method, setting, target population, practitioner qualifications, scheduling, or referrals. Moreover, only three websites included patient testimonials even though testimonials have been shown to affect health behavior change [64-67]. Similarly, only five websites included information on the cost of MBIs despite costs being a well-described factor when considering the use of complementary therapies [68,69].

CCs should continually evaluate their websites for scope, findability, and quality of MBI-related information. Behavioral frameworks, such as the TPB, can help identify the key pieces of information that drive patients’ treatment decision-making and their willingness to seek MBIs [70]. We recommend that CCs have a dedicated web page for MBI that is easily findable from the CC home page and includes the following information:

- Brief descriptions of MBIs, what patient participation involves, and specificity regarding whether interventions are offered individually or in group.
- Clinical indications or treatment benefits of MBIs with references to research literature and links to evidence-based resources (eg, American Music Therapy Association or Cochrane Library).
- Target populations, location of services, and method of delivery (in person or remote).
- Details about the qualifications and training of MBI practitioners, particularly those who are not board-certified music therapists.
- Costs of service.
- Instructions for scheduling MBI appointments or referring patients to MBIs.
- Testimonials by patients or health care providers, including example videos of patients engaging in MBIs.

While websites represent a key source of information that may drive treatment-seeking behavior, it is also important to consider other factors (eg, health care providers’ knowledge and beliefs) that may influence the use of MBIs. For example, one study found that only 56% of health care providers in oncology settings knew about the role of MT in cancer symptom management and knew how to make referrals to MT [71]. Since most patients with cancer look to their primary oncology team to provide information about complementary or integrative health options [72], future dissemination efforts should focus on targeting health care professionals’ knowledge about MBIs and establishing clear pathways for referring patients to MBIs. Fostering more seamless interprofessional collaboration between music therapists and other health care providers may also help to increase knowledge of MBIs and promote greater uptake of MBIs in oncology settings [73].

### Limitations

Our study has some limitations. First, the time period of data extraction may not reflect the current website landscape. Next, descriptions on the websites may not be indicative of real-world availability of MBIs at the NCI-designated CCs. Additionally, the search strategy we employed was limited to the websites made publicly available by the CCs. Several CCs were also housed within multiple hospitals. Therefore, we may have missed some descriptions of MBIs. Furthermore, CCs may have not updated their websites with their current MBI offerings. In addition, the generalizability of our findings may be limited because we only searched the websites of NCI-designated CCs, all of which are located in the United States. Finally, a validated scale for rating the quality of MBI-related information found on websites does not exist in the literature, so we adapted a rating scale that other researchers used to evaluate public-facing websites of NCI-designated CCs [41]. While the findings should be interpreted as preliminary, our study could potentially inform future research to develop a rigorous, validated scale for rating health information found on public-facing websites.

### Conclusions

Despite these limitations, our study is the first to provide a comprehensive review of MBI-related information on public-facing websites at NCI-designated CCs. NCI-designated CCs are often viewed by patients, families, and community providers as the gold standard for sources of information. In an increasingly digital world, it is critical for NCI-designated CCs to maintain a robust online presence and update their public-facing websites with evidence-informed information about MBIs. As the evidence for MBIs grows, translation of this research into accessible, actionable knowledge is critical to the real-world delivery and use of MBIs. While our study showcases the wide range of MBIs described in various oncology settings, the findings also highlight that NCI-designated CCs need to provide more detailed information about MBIs on their public-facing websites to promote the dissemination and implementation of this evidence-based option for cancer symptom management [20].

In addition to NCI-designated CC websites, future research should evaluate other key avenues through which information about MBIs is disseminated to the public. Most patients with cancer receive care in community settings [74], so it will be important to research how MBI-related information is presented in community-based clinics, hospitals, and cancer advocacy organizations. Music represents a multicultural resource [35,36], and research on the use of MBIs in cancer care has been conducted in various countries around the world [20]. Future studies should examine how MBIs are presented to the public in this global context. Researchers should also strive to develop culturally attuned approaches to disseminating MBI-related information so that patients with cancer from all cultures and backgrounds could learn about this evidence-based modality.

## Abbreviations

CC: Cancer Center
CCC: Comprehensive Cancer Center
MBI: music-based intervention
MS: music services
MT: music therapy
NCI: National Cancer Institute
TPB: Theory of Planned Behavior

## References

[1] National Cancer Institute (2015). 2015 strategic priorities: symptom management & quality of life steering committee (SxQoL SC). National Cancer Institute.

[2] Greco MT, Roberto A, Corli O (2014). Quality of cancer pain management: an update of a systematic review of undertreatment of patients with cancer. J Clin Oncol.

[3] Erdoğan Yüce G, Döner A, Muz G (2021). Psychological distress and its association with unmet needs and symptom burden in outpatient cancer patients: a cross-sectional study. Semin Oncol Nurs.

[4] Firkins J, Hansen L, Driessnack M, Dieckmann N (2020). Quality of life in “chronic” cancer survivors: a meta-analysis. J Cancer Surviv.

[5] Hershman DL, Shao T, Kushi LH (2011). Early discontinuation and non-adherence to adjuvant hormonal therapy are associated with increased mortality in women with breast cancer. Breast Cancer Res Treat.

[6] Nipp RD, El‐Jawahri A, Moran SM (2017). The relationship between physical and psychological symptoms and health care utilization in hospitalized patients with advanced cancer. Cancer.

[7] Haskins CB, McDowell BD, Carnahan RM (2019). Impact of preexisting mental illness on breast cancer endocrine therapy adherence. Breast Cancer Res Treat.

[8] Davis NE, Hue JJ, Kyasaram RK (2022). Prodromal depression and anxiety are associated with worse treatment compliance and survival among patients with pancreatic cancer. Psychooncology.

[9] Chim K, Xie SX, Stricker CT (2013). Joint pain severity predicts premature discontinuation of aromatase inhibitors in breast cancer survivors. BMC Cancer.

[10] Murphy CC, Fullington HM, Alvarez CA (2018). Polypharmacy and patterns of prescription medication use among cancer survivors. Cancer.

[11] Vyas A, Alghaith G, Hufstader-Gabriel M (2020). Psychotropic polypharmacy and its association with health-related quality of life among cancer survivors in the USA: a population-level analysis. Qual Life Res.

[12] Choi DW, Kang H, Zhang HS, Jhang H, Jeong W, Park S (2023). Association of polypharmacy with all-cause mortality and adverse events among elderly colorectal cancer survivors. Cancer.

[13] Mohamed MR, Mohile SG, Juba KM (2023). Association of polypharmacy and potential drug-drug interactions with adverse treatment outcomes in older adults with advanced cancer. Cancer.

[14] Berger AM, Mooney K, Alvarez-Perez A (2015). Cancer-related fatigue, version 2.2015. J Natl Compr Canc Netw.

[15] Andersen BL, DeRubeis RJ, Berman BS (2014). Screening, assessment, and care of anxiety and depressive symptoms in adults with cancer: an American Society of Clinical Oncology guideline adaptation. J Clin Oncol.

[16] Romero SAD, Brown JC, Bauml JM (2018). Barriers to physical activity: a study of academic and community cancer survivors with pain. J Cancer Surviv.

[17] Eylem O, de Wit L, van Straten A (2020). Stigma for common mental disorders in racial minorities and majorities a systematic review and meta-analysis. BMC Public Health.

[18] McGuire TG, Miranda J (2008). New evidence regarding racial and ethnic disparities in mental health: policy implications. Health Aff (Millwood).

[19] Bradt J, Potvin N, Kesslick A (2015). The impact of music therapy versus music medicine on psychological outcomes and pain in cancer patients: a mixed methods study. Support Care Cancer.

[20] Bradt J, Dileo C, Myers-Coffman K, Biondo J (2021). Music interventions for improving psychological and physical outcomes in people with cancer. Cochrane Database Syst Rev.

[21] (1998). AMTA official definition of music therapy. American Music Therapy Association.

[22] Dileo C (2013). A proposed model for identifying practices: a content analysis of the first 4 years of Music and Medicine. Mus Med.

[23] (2023). NCCN guidelines: treatment by cancer type. National Comprehensive Cancer Network.

[24] Greenlee H, DuPont-Reyes MJ, Balneaves LG (2017). Clinical practice guidelines on the evidence-based use of integrative therapies during and after breast cancer treatment. CA Cancer J Clin.

[25] Carlson LE, Ismaila N, Addington EL (2023). Integrative oncology care of symptoms of anxiety and depression in adults with cancer: Society for Integrative Oncology-ASCO guideline. J Clin Oncol.

[26] Carlson LE, Ismaila N, Addington EL (2023). Integrative oncology care of symptoms of anxiety and depression in adults with cancer: SIO-ASCO guideline summary and Q&A. JCO Oncol Pract.

[27] Koelsch S (2014). Brain correlates of music-evoked emotions. Nat Rev Neurosci.

[28] Overy K, Molnar-Szakacs I (2009). Being together in time: musical experience and the mirror neuron system. Music Percept.

[29] Finn S, Fancourt D (2018). The biological impact of listening to music in clinical and nonclinical settings: a systematic review. Prog Brain Res.

[30] Chanda ML, Levitin DJ (2013). The neurochemistry of music. Trends Cogn Sci.

[31] McPherson T, Berger D, Alagapan S, Fröhlich F (2019). Active and passive rhythmic music therapy interventions differentially modulate sympathetic autonomic nervous system activity. J Music Ther.

[32] Mas-Herrero E, Dagher A, Farrés-Franch M, Zatorre RJ (2021). Unraveling the temporal dynamics of reward signals in music-induced pleasure with TMS. J Neurosci.

[33] Menon V, Levitin DJ (2005). The rewards of music listening: response and physiological connectivity of the mesolimbic system. Neuroimage.

[34] Zatorre RJ, Salimpoor VN (2013). From perception to pleasure: music and its neural substrates. Proc Natl Acad Sci U S A.

[35] Mehr SA, Singh M, Knox D (2019). Universality and diversity in human song. Science.

[36] Savage PE, Brown S, Sakai E, Currie TE (2015). Statistical universals reveal the structures and functions of human music. Proc Natl Acad Sci U S A.

[37] Finney Rutten LJ, Blake KD, Greenberg-Worisek AJ, Allen SV, Moser RP, Hesse BW (2019). Online health information seeking among US adults: measuring progress toward a Healthy People 2020 objective. Pub Health Rep.

[38] van Eenbergen M, Vromans RD, Boll D (2020). Changes in internet use and wishes of cancer survivors: a comparison between 2005 and 2017. Cancer.

[39] Vlooswijk C, Husson O, Krahmer EJ (2021). Differences in internet use and eHealth needs of adolescent and young adult versus older cancer patients; results from the PROFILES Registry. Cancers (Basel).

[40] Rolland B, Eschler J (2018). Searching for survivor-specific services at NCI-designated comprehensive cancer centers: a qualitative assessment. J Natl Compr Canc Netw.

[41] Silver JK, Raj VS, Fu JB (2018). Most national cancer institute-designated cancer center websites do not provide survivors with information about cancer rehabilitation services. J Canc Educ.

[42] Wechsler S, Ma M, El-Jawahri A (2024). Employment-related education and support for cancer survivors: a content analysis of employment resources offered on National Cancer Institute-designated cancer center websites. J Canc Educ.

[43] Geissler KH, Evans V, Cooper MI, Shaw SJ, Yarrington C, Attanasio LB (2023). Content analysis of patient-facing information related to preeclampsia. Womens Health Issues.

[44] Huxley K, Atkinson P, Delamont S, Cernat A, Sakshaug JW, Williams RA (2020). SAGE Research Methods Foundations.

[45] (2023). Find a cancer center. National Cancer Institute.

[46] Desai K, Liou K, Liang K, Seluzicki C, Mao JJ (2021). Availability of integrative medicine therapies at National Cancer Institute-designated comprehensive cancer centers and community hospitals. J Altern Complement Med.

[47] Yun H, Sun L, Mao JJ (2017). Growth of integrative medicine at leading cancer centers between 2009 and 2016: a systematic analysis of NCI-designated comprehensive cancer center websites. J Natl Cancer Inst Monogr.

[48] Ajzen I (2011). The theory of planned behaviour: reactions and reflections. Psychol Health.

[49] Hirai K, Komura K, Tokoro A (2008). Psychological and behavioral mechanisms influencing the use of complementary and alternative medicine (CAM) in cancer patients. Ann Oncol.

[50] Bauml JM, Chokshi S, Schapira MM (2015). Do attitudes and beliefs regarding complementary and alternative medicine impact its use among patients with cancer? A cross-sectional survey. Cancer.

[51] Perez SL, Kravitz RL, Bell RA, Chan MS, Paterniti DA (2016). Characterizing internet health information seeking strategies by socioeconomic status: a mixed methods approach. BMC Med Inform Decis Mak.

[52] Perez SL, Paterniti DA, Wilson M (2015). Characterizing the processes for navigating internet health information using real-time observations: a mixed-methods approach. J Med Internet Res.

[53] (2023). Music for Healing & Transition Program.

[54] George GC, Iwuanyanwu EC, Buford AS (2019). Cancer-related internet use and its association with patient decision making and trust in physicians among patients in an early drug development clinic: a questionnaire-based cross-sectional observational study. J Med Internet Res.

[55] Li Y, Xing X, Shi X (2020). The effectiveness of music therapy for patients with cancer: a systematic review and meta‐analysis. J Adv Nurs.

[56] Murakami B (2021). The music therapy and harm model (MTHM). Conceptualizing harm within music therapy practice. Rev Cient Musicoter Discip Afines.

[57] Gupta A, Eisenhauer EA, Booth CM (2022). The time toxicity of cancer treatment. J Clin Oncol.

[58] Cheng AC, Levy MA (2019). Measures of treatment workload for patients with breast cancer. JCO Clin Cancer Inform.

[59] Yabroff KR, Davis WW, Lamont EB (2007). Patient time costs associated with cancer care. J Natl Cancer Inst.

[60] Yabroff KR, Guy GP, Ekwueme DU (2014). Annual patient time costs associated with medical care among cancer survivors in the United States. Med Care.

[61] Yabroff KR, Mariotto A, Tangka F (2021). Annual report to the nation on the status of cancer, part 2: patient economic burden associated with cancer care. J Natl Cancer Inst.

[62] Rocque GB, Williams CP, Miller HD (2019). Impact of travel time on health care costs and resource use by phase of care for older patients with cancer. J Clin Oncol.

[63] Rocque GB, Williams CP, Ingram SA (2020). Health care-related time costs in patients with metastatic breast cancer. Cancer Med.

[64] Dillard AJ, Main JL (2013). Using a health message with a testimonial to motivate colon cancer screening: associations with perceived identification and vividness. Health Educ Behav.

[65] Dillard AJ, Ferrer RA, Welch JD (2018). Associations between narrative transportation, risk perception and behaviour intentions following narrative messages about skin cancer. Psychol Health.

[66] Shaffer VA, Brodney S, Gavaruzzi T (2021). Do personal stories make patient decision aids more effective? An update from the international patient decision aids standards. Med Decis Making.

[67] Woudstra AJ, Suurmond J (2019). How narratives influence colorectal cancer screening decision making and uptake: a realist review. Health Expect.

[68] Khan HM, Ramsey S, Shankaran V (2023). Financial toxicity in cancer care: implications for clinical care and potential practice solutions. J Clin Oncol.

[69] Smith GL, Banegas MP, Acquati C (2022). Navigating financial toxicity in patients with cancer: a multidisciplinary management approach. CA Cancer J Clin.

[70] Jia X, Pang Y, Liu LS (2021). Online health information seeking behavior: a systematic review. Healthcare (Basel).

[71] Esplen MJ, Foster B, Pearson S (2020). A survey of oncology healthcare professionals’ knowledge and attitudes toward the use of music as a therapeutic tool in healthcare. Supp Care Cancer.

[72] Bari S, Chineke I, Darwin A (2021). Awareness, use and outlook of complementary and alternative medicine (CAM) options in an underserved, uninsured minority cancer patient population. Integr Cancer Ther.

[73] Lacson C (2022). Interprofessional collaboration between pediatric music therapists and multidisciplinary team members in hospitals: an explanatory sequential mixed methods study. Dissertation.

[74] Hughes-Halbert C (2017). Bringing research to the community to reduce cancer disparaties. National Cancer Institute.

